# Evidence for a Cyanine Link Between Propargylamine Drugs and Monoamine Oxidase Clarifies the Inactivation Mechanism

**DOI:** 10.3389/fchem.2018.00169

**Published:** 2018-05-28

**Authors:** Alen Albreht, Irena Vovk, Janez Mavri, Jose Marco-Contelles, Rona R. Ramsay

**Affiliations:** ^1^Department of Food Chemistry, National Institute of Chemistry, Ljubljana, Slovenia; ^2^Laboratory of Computational Biochemistry and Drug Design, Theory Department, National Institute of Chemistry, Ljubljana, Slovenia; ^3^Laboratorio de Química Médica, Instituto de Química Orgánica General (CSIC), Madrid, Spain; ^4^Biomedical Sciences Research Complex, University of St Andrews, St Andrews, United Kingdom

**Keywords:** monoamine oxidase, propargylamine, inhibition mechanism, structure, electrostatic interactions, quantum chemical calculations, isomers, interconversion

## Abstract

Successful propargylamine drugs such as deprenyl inactivate monoamine oxidase (MAO), a target in multi-faceted approaches to prevent neurodegeneration in the aging population, but the chemical structure and mechanism of the irreversible inhibition are still debated. We characterized the covalent cyanine structure linking the multi-target propargylamine inhibitor ASS234 and the flavin adenine dinucleotide in MAO-A using a combination of ultra-high performance liquid chromatography, spectroscopy, mass spectrometry, and computational methods. The partial double bond character of the cyanine chain gives rise to 4 interconverting geometric isomers of the adduct which were chromatographically separated at low temperatures. The configuration of the cyanine linker governs adduct stability with segments of much higher flexibility and rigidity than previously hypothesized. The findings indicate the importance of intramolecular electrostatic interactions in the MAO binding site and provide key information relevant to incorporation of the propargyl moiety into novel multi-target drugs. Based on the structure, we propose a mechanism of MAO inactivation applicable to all propargylamine inhibitors.

## Introduction

**Figure 1:**
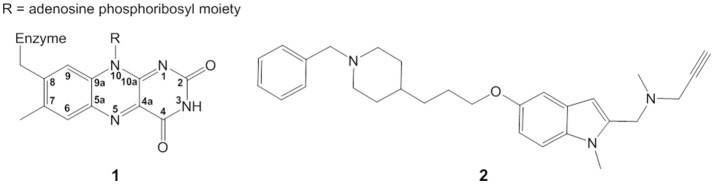
Structure of isoalloxazine ring **(1)** and ASS234 propargylamine inhibitor **(2)**.

Monoamine oxidases (MAO-A and MAO-B) catalyze the oxidative deamination of monoamine neurotransmitters such as dopamine and serotonin (Weyler et al., 1990). The isoalloxazine ring (Figure 1) of the covalently attached FAD moiety **(1)** is directly involved in the transformation of primary, secondary, and tertiary amines into positively charged imines with concomitant reduction of FAD to FADH^−^. Imines are afterwards non-enzymatically hydrolyzed to yield the corresponding aldehyde and amine (Edmondson et al., 1993; Woo and Silverman, 1995). Despite much discussion, the catalytic mechanism is still debated (Silverman et al., 1980; Silverman, 1995b; Miller and Edmondson, 1999; Edmondson et al., 2004b, 2009; Li et al., 2006; Vianello et al., 2012; Akyüz and Erdem, 2013; Orru et al., 2013; Pavlin et al., 2013; Chajkowski-Scarry and Rimoldi, 2014; Repič et al., 2014). The oxidation of the amine is also the first step of the irreversible inhibition pathway by several types of inactivating compounds (Silverman, 1995a; Kalgutkar et al., 2001; Chajkowski-Scarry and Rimoldi, 2014). Although the inhibition of MAO by propargylamine derivatives used in the treatment of neuropsychiatric and age-related neurodegenerative disorders (Yu, 1994; Youdim et al., 2006) dates to the late 1950s (Taylor et al., 1960), the structure of irreversible propargylamine-inhibited MAO and the mechanism of formation are still not fully understood (Edmondson et al., 2009; Pavlin et al., 2013). Elucidating the details of the chemical mechanism of MAO inactivation is key to rational design of new generation drugs.

Plausible covalent adduct structures and inhibition mechanisms of MAO by propargylamines were proposed by Maycock et al. in 1975 (Maycock, 1975; Maycock et al., 1976a,b). Proposed mechanisms without experimental evidence included: (i) an enzymatic abstraction of the propargylic proton from the inhibitor and its subsequent attack on oxidized FAD, (ii) a mechanism proceeding through radical intermediates which then collapse to form the cyanine adduct, (iii) a reduced FAD moiety reacting with an oxidized inhibitor into the covalent adduct. Later, in model reactions Nakai et al. employed truncated analogs of isoalloxazine and (–)-deprenyl in quantum chemical investigations (Nakai et al., 1999) to predict the formation of two stable cyclic adducts (O4,N5- and C4a,N5-adduct) formed through a series of intermediates and transition states. In a computational study, Borštnar et al. proposed the deprotonated acetylenic moiety of propargylamine as the reactive species to attack the electrophilic N(5) of the oxidized flavin (Borštnar et al., 2011), resulting in an adduct bearing an alkynyl moiety. The inhibition mechanisms from these studies are inconclusive. Furthermore, for the covalent binding of a propargylamine inhibitor to MAO, adduct structures from different studies are inconsistent (Gärtner et al., 1976; Nakai et al., 1999; Kalgutkar et al., 2001; Binda et al., 2002, 2004; Edmondson et al., 2004b; Hubálek et al., 2004; Borštnar et al., 2011; Pavlin et al., 2013; Esteban et al., 2014).

ASS234 is a multi-target propargylamine compound that inhibits both MAO and cholinesterases (Samadi et al., 2012). It also has antioxidant capacity and prevents beta-amyloid aggregation, making it a lead compound for Alzheimer's disease treatment (Marco-Contelles et al., 2016). Here we elucidate the structure of the covalent adduct between MAO-A and the multi-target propargylamine ASS234 **(2)** (Figure 1) by digesting the inhibited enzyme to the flavopentapeptide (Nagy and Salach, 1981), and separating and characterizing it by ultra high-performance liquid chromatography coupled to photodiode array and MS detectors (UHPLC-PDA-MS). Supported by quantum chemical calculations and stopped-flow spectrometry, we propose a general mechanism for MAO inactivation by propargylamine drugs.

## Material and methods

The propargylamine inhibitor ASS234 was synthesized as before (Samadi et al., 2012).

### Inactivation of MAO-A with ASS234 and enzymatic digestion

Recombinant human MAO-A was purified (Weyler and Salach, 1985; Hynson et al., 2003) and dialyzed against 50 mM HEPES pH 7.5 for 3 h. For the inactivation, MAO-A (40 μM) was incubated with ASS234 (200 μM) at 30°C and monitored spectrophotometrically to completion [Shimadzu 2101PC (Kyoto, Japan) or PerkinElmer Lambda 45 (Waltham, MA, USA)]. For digestion by trypsin/chymotrypsin (0.02 mg of each per mg of MAO protein), the solution was incubated at 37°C for 3 h. Another aliquot of trypsin/chymotrypsin was added and incubated overnight. The digestion was quenched by the addition of HCOOH and stored at −20°C prior to UHPLC-PDA-MS^n^ analyses. The same procedure was followed for the control sample without inhibitor and when MAO-A was inhibited by clorgyline. Additional spectroscopic details are provided in Supplementary Material.

For kinetics, MAO-A (40 μM) was mixed with an equal volume of 50 mM HEPES pH 7.5 containing ASS234 (120 μM) in the stopped-flow spectrophotometer (Applied Photophysics SX20) at 30°C. Triplicate runs at three wavelengths (410, 456, and 495 nm) were analyzed using single or double exponential fits in the built-in software. Additional details are provided in Supplementary Material.

### UHPLC-PDA-MS^n^ analyses

The UHPLC system Accela 1250 with a PDA detector (Thermo Finnigan, San Jose, CA, USA) was coupled to the LTQ Velos MS system (Thermo Finnigan) equipped with a heated electrospray ionization source (H-ESI). Xcalibur (2.1) software was used for evaluation of the collected data. Further experimental and instrument details are provided in Supplementary Material.

### Detection of geometric isomers with varying temperature

The chromatographic and MS parameters were unaltered; only the temperature was varied between −10, 10, 30, 45, 60 and 75°C. The fractionation of 2 isomers was achieved at 10°C by injecting 25 μL of MAO-A-ASS234 digest solution (4 μM) into the column and collecting each individual peak separately at the PDA exit. The process was repeated 5 times. The total volume of both fractions was brought down to 50 μL and 25 μL of each fraction was re-analyzed separately by UHPLC at 30°C. Additional experimental details are provided in Supplementary Material.

### Computational methods

The Gibbs activation energy (Δ*G*^‡^) for the *cis-trans* isomerization of the cyanine chain was determined from experimental chromatographic data using transition state theory. The interconversion energy barrier was calculated by the Eyring-Polanyi equation describing the relation between the reaction rate and the activation free energy:

$$\begin{array}{l} k=\frac{k_{B}T}{h}e^{-\frac{\Delta G^{‡}}{RT}} \end{array}$$

where *k* = reaction rate constant, *k*_*B*_ = Boltzmann constant, *h* = Planck's constant, *T* = temperature, and *R* = gas constant. The Δ*G*^‡^ for the reaction at a given temperature is calculated from the rate constant. Rate constants for the isomerization observed in chromatograms at a particular temperature were determined by DCXplorer software (Trapp, 2006). Rate constants and Δ*G*^‡^ values were determined from the experimental chromatographic data at 10 and 30°C.

### Quantum chemical calculations of internal rotations of cyanine chain

The initial structure of the studied pentapeptide-FAD-ASS234 adduct was obtained by model building in Molden (Schaftenaar and Noordik, 2000). A truncated model of the adduct was used since the adenosine phosphoribosyl, pentapeptide moieties from MAO-A and the cholinesterase inhibiting part of ASS234 were assumed to have no significant contribution on the configuration of the cyanine chain. Initially unconstrained energy minimization of the adduct was performed. Several starting geometries were used in order to find the global minimum from which we started internal rotation energy profile calculations by the procedure as follows: four internal rotations were considered and energy profiles were calculated by using a relaxed scan. The starting geometry for each dihedral angle was the optimized geometry of preceding dihedral value. Structures corresponding to the profiles of internal rotations are formally conditional minima with one fixed value of the dihedral angle with all other degrees of freedom optimized. Therefore, rotation profiles obtained by this procedure correspond to the minimum energy path. Calculations were performed on the M06-2x/6-311+G (d,p) level using the Gaussian 09 suite of programs (Frisch et al., 2009). For energy minimization default values for convergence criteria were used as built in Gaussian 09. Some values of dihedral angles resulted in steric repulsion giving rise to very high energies and the geometry optimization procedure was automatically terminated. We did not report rotation profiles for those dihedral angles values. Preliminary results indicated that solvent has only minor effect on energy profiles and was therefore omitted in the calculations.

## Results and discussion

### MAO-A-ASS234 adduct formation

**Figure 2:**
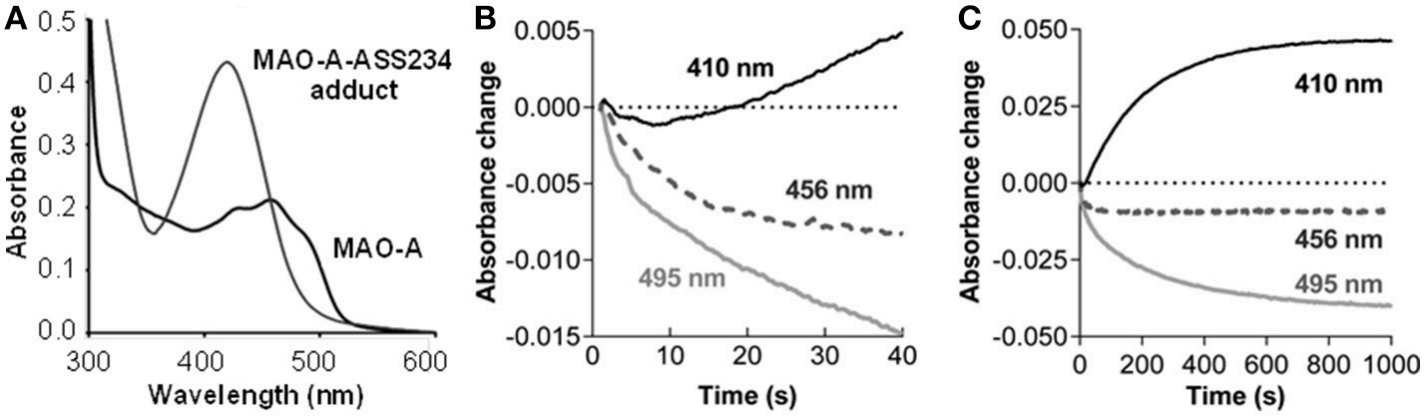
ASS234 inhibition of MAO-A. **(A)** Spectra for MAO-A before and after ASS234 inactivation. **(B)** and **(C)** Absorbance changes for the first 40 s and 1000 s, respectively, at 410, 456, and 495 nm after rapid mixing of equal volumes of MAO-A (40 μM) and ASS234 (120 μM) at 30°C in a stopped-flow spectrophotometer.

Inactivation of MAO-A by the propargylamine ASS234 was accompanied by changes in the UV-VIS spectrum (Figure 2A). The absorption maximum at physiological conditions shifted from 456 nm (oxidized FAD) to 410 nm in parallel with loss of enzyme activity and formation of an adduct between MAO-A and ASS234 (MAO-A-ASS234). When the adduct-containing solution was acidified (pH 1) the absorption maximum shifted toward 370 nm (Figure S1, Supplementary Material). This hypsochromic shift confirms the N(5) adduct formation whereas a bathochromic shift would indicate the C(4a) adduct (Ghisla et al., 1973; Gärtner and Hemmerich, 1975).

The inactivation process was also monitored in the stopped-flow spectrophotometer at three wavelengths: 456 nm—an isosbestic point of the reaction in the steady-state; 495 nm—a selective wavelength for flavin reduction (i.e., the enzyme catalyzed reaction); and 410 nm—a wavelength for the adduct formation. After rapid mixing of MAO-A and ASS234, there was a small decrease in absorption at 456 nm before the isosbestic point was established (Figure 2B). The rate of the initial decrease at 495 nm shown in Figures 2B,C is 0.057 ± 0.006 s^−1^ (*n* = 3), similar to the rate of the pre-steady-state change at 456 nm [*k* = 0.056 ± 0.006 s^−1^ (*n* = 3)]. At 410 nm where the adduct absorbs strongly, a small decrease is observed initially, but the absorbance starts to increase after 8 s with a 10-fold slower rate of 0.005 ± 0.006 s^−1^ (*n* = 3), similar to the steady-state rate of inactivation (Esteban et al., 2014). This distinct lag before product is detected is a pattern typical for the second reaction in a kinetic sequence, indicating that FAD reduction precedes the formation of the covalent adduct as has been observed previously for rasagiline and its analogs (Hubálek et al., 2004).

### MAO-A-ASS234 adduct pentapeptide characterization

In the UHPLC-DAD-MS analysis of the tryptic/chymotryptic digest the uninhibited MAO-A gave a single sharp chromatographic peak at 6.9 min with strong absorbance at 450 nm (Figure S2) and an MS signal at *m/z* 1267 corresponding to the [M–H]^−^ of FAD-SGGCY pentapeptide (Figure S3). Tandem MS spectrum, using a precursor ion at *m/z* 1267, showed a strong fragment ion at *m/z* 920 (Figure S4) as a result of adenosine monophosphate (AMP) cleavage from FAD-pentapeptide. This neutral loss of 347 was used in subsequent analyses as an identifier of FAD moiety.

**Figure 3:**
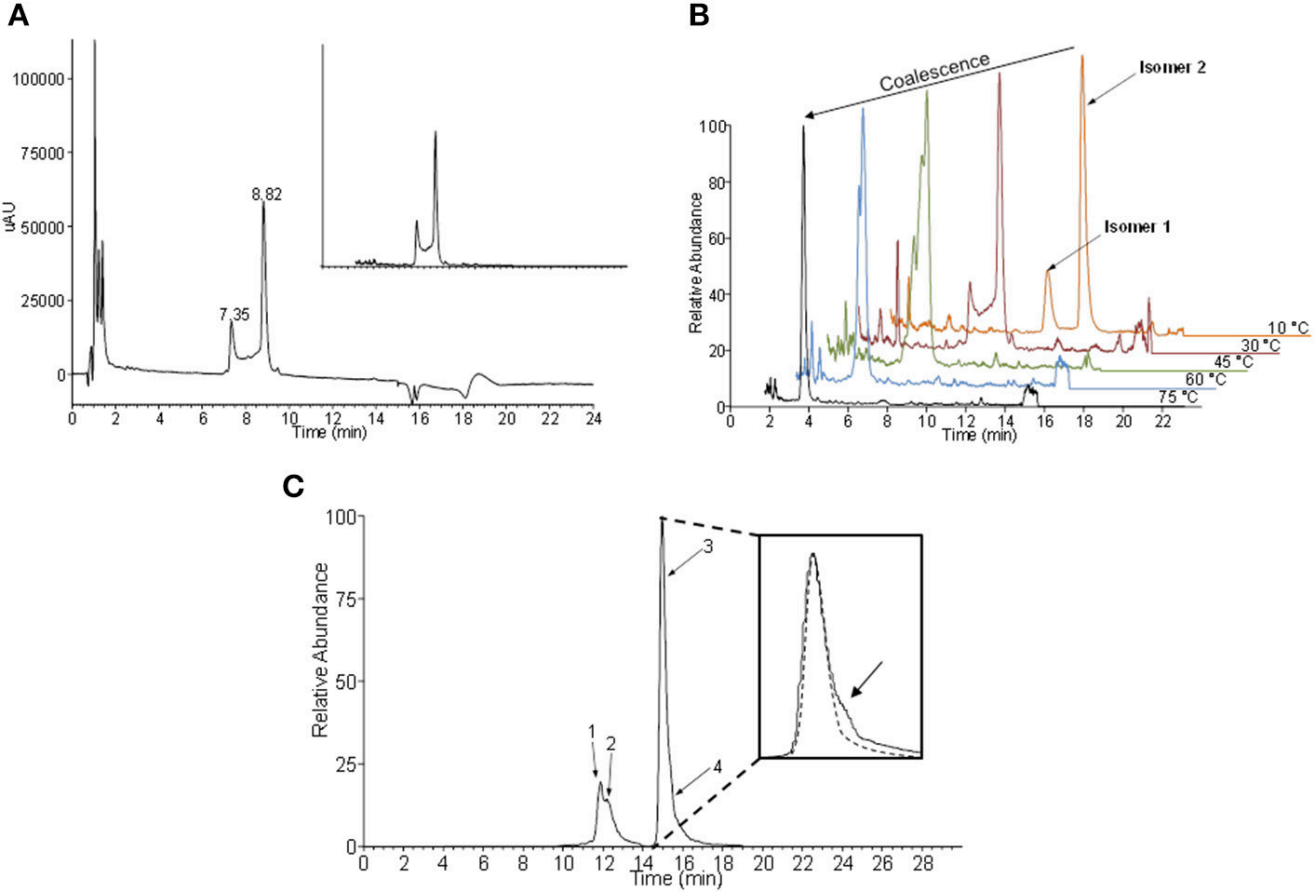
Geometric isomers of the flavin-ASS234 adduct from tryptic/chymotryptic digested MAO-A. **(A)** UHPLC chromatogram of ASS234-inhibited MAO-A digest acquired at 410 nm (inset—extracted ion chromatogram at *m/z* 1710). **(B)** Temperature dependence of the interconversion rate for two geometric isomers. At high temperatures isomers coalesce into a single chromatographic peak. **(C)** The extracted ion chromatogram at *m/z* 1710 obtained at −10°C indicates the existence of at least four isomers of the covalent adduct. A low abundant fourth isomer was detected as a shoulder on the major chromatographic peak (inset)—dashed line simulates the expected shape of the major peak without co-elution.

**Figure 4:**
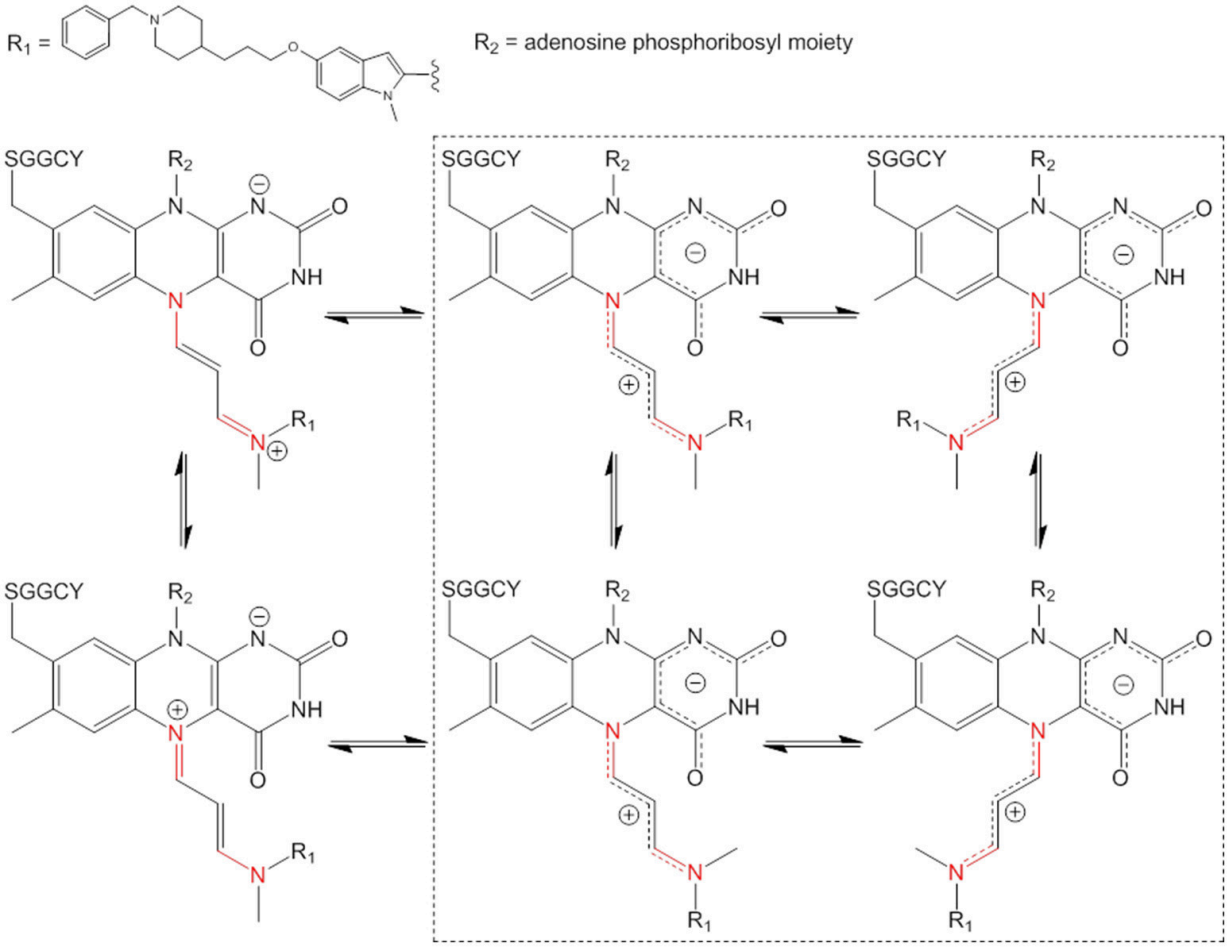
Some examples of isomeric structures of the pentapeptide-FAD-ASS234 covalent adduct which can be formed by intramolecular rotations only about C–N bonds (in red). Both positive charge delocalization extremes of the cyanine chain are depicted on the left hand side. The four possible geometric structures are highlighted in the dashed square (intramolecular rotations about cyanine C–C bonds are not considered).

The analysis of ASS234-inactivated MAO-A digest (Figure 3A) gave a pair of peaks at 7.4 and 8.8 min which exhibited reduced absorbance at 450 nm. The absorption spectra of these two peaks are identical, with λ_max_ at 395 nm. The absorption maximum for the peptide adduct is slightly blue-shifted from the 410 nm observed in the intact MAO-A-ASS234 (physiological conditions) (Figure 2) due to mobile phase solvent and pH effects. There is also a plateau between the peaks, which is not observed in the uninhibited MAO-A digest. Even though this pattern is not characteristic of fronting/tailing, these and some other common chromatographic artifacts were nevertheless systematically ruled out (Discussion S1), leaving as its cause an intrinsic structural feature of the adduct after inactivation of MAO-A by ASS234. The MS spectra of both peaks were identical and showed a signal at *m/z* 1710, corresponding to the pentapeptide-FAD-ASS234 covalent adduct (Figure S5). Moreover, the extracted ion chromatogram at *m/z* 1710 perfectly overlapped with the 410 nm UV trace and showed the same plateau between the two peaks (Figure 3A inset). Supporting the presence of FAD moiety in the precursor ion at *m/z* 1710, MS^2^ gave a fragment ion at *m/z* 1363, obtained by the neutral loss of AMP (347 amu) (Figure S6). We hypothesized that the two “bridged” peaks with exactly the same absorption and mass spectra are geometric or conformational isomers. Representations of some possible adduct structures are depicted in Figure 4, with the underpinning reasoning and experimental confirmation given below. An all-*trans* planar configuration of the covalent linker between the enzyme and the inhibitor (cyanine chain) was assumed at first, because the ground state of such a system generally results in higher resonance energy stabilization (Brooker et al., 1947; West et al., 1967). Moreover, the cyanine chain bears no bulky substituents which would cause steric hindrance and adaptation of an alternative configuration.

Conclusively, the observed signal for deprotonated adduct molecule at *m/z* 1710 resolves the previous ambiguity of the form of the reduced flavin in MAO-A (Edmondson et al., 2004a)—it is the anionic form (FADH^−^). The pK_a_ of N(1) in the flavin peptide is substantially lower than in the native MAO-A as it remains deprotonated even under our fairly acidic experimental conditions (pH ≈ 2). MS data also prove the covalent bond between MAO-A and ASS234 (see Discussion S2 for further insight).

### MAO-A-ASS234 adduct structure elucidation

The electronic structure of the adduct is complex, but it is reasonable to speculate that the positive charge is delocalized within the cyanine chain which has mixed single and double bond character (Figure 4). Therefore, the conjugated scaffold should be reflected in hindered rotation about both pairs of C–N and C–C bonds. Internal rotations of such systems are generally additionally disfavored because the resonance stabilization of the positive charge is interrupted by the transition state between the two forms and this should particularly be true for rotations about C–C bonds in the middle of the cyanine chain. Our assumption of a dynamic geometric isomerism was demonstrated by the experiment shown in Figure 3B. Two adduct forms were separated chromatographically at 10°C as two clearly distinguished peaks because at this temperature the rate of *cis-trans* isomerization was sufficiently low. Quantum-chemically calculated energy profiles for internal rotations suggest that the structure of the adduct is semi-flexible and associated with several minima that are separated with the barriers of about 20 kcal/mol (see below). As the temperature of the chromatographic analysis increased, the two isomers interchanged more rapidly until, at the highest temperature (75°C), only one peak was detected. The same phenomenon was observed when ASS234 was exchanged for clorgyline, a standard propargylamine inactivator of MAO-A, but the interconversion barrier was higher so that the isomers were well separated even at higher temperatures (Figure S7).

Additional confirmation of the existence of interconverting geometric isomers for the ASS234 adduct was obtained by collecting the peaks at 10°C as two individual fractions from the UHPLC column outlet. The collected fractions were re-analyzed by UHPLC, only to find that the adduct was again seen as a mixture of two species in both cases (data not shown). The “bridge” between peaks in Figure 3A can be ascribed to the individual molecules which underwent at least one interconversion cycle during the UHPLC experiment. Since the cyanine chain consists of 2 non-equivalent pairs of C–N and C–C bonds (Figure 4) there are 16 (2^4^) hypothetically viable geometric isomers if we allow deviations from a theoretically more stable all-*trans* configuration. However, not all, but only isomers with a low enough molecular potential energy (stable isomers) can actually be detected. By significantly lowering the temperature in the chromatographic experiment (to −10°C), two additional species were experimentally confirmed (Figure 3C), which indirectly demonstrates the existence of the cyanine chain in the adduct. Due to its low abundance, the fourth isomer was detected merely as a shoulder of the main peak (Figure 3C—inset). Chromatographic tailing could be ruled out as a probable cause of this observation since tailing peaks do not contain an inflection point (shoulder) unless there is co-elution of one or more compounds. We were unable to detect any further isomers because use of temperatures lower than −10°C in the experiment was not possible.

The isomerization rate constants at two temperatures (10°C, 30°C) were determined from our raw experimental chromatographic data using DCXplorer software [based on the unified equation of chromatography (Trapp, 2006)]. Then, the interconversion energy barrier (Δ*G*^‡^) for the two isomers clearly separated at 10°C was calculated by employing the transition state theory (Eyring-Polanyi equation). The rate constant at 10°C was 2.1 × 10^−4^ s^−1^ yielding Δ*G*^‡^ = 21.3 kcal/mol. At 30°C the rate constant increased to 2.0 × 10^−3^ s^−1^ corresponding to Δ*G*^‡^ = 21.5 kcal/mol. With clorgyline at 30°C, a lower rate constant was obtained (3.9 × 10^−4^ s^−1^) and the activation energy proportionately higher (Δ*G*^‡^ = 23.6 kcal/mol). The overall activation energies (Δ*G*^‡^ ≈22 kcal/mol) for the isomerization in these adducts are similar to rotation energy barriers for C–N amide bonds possessing partial double-bond character.

**Figure 5:**
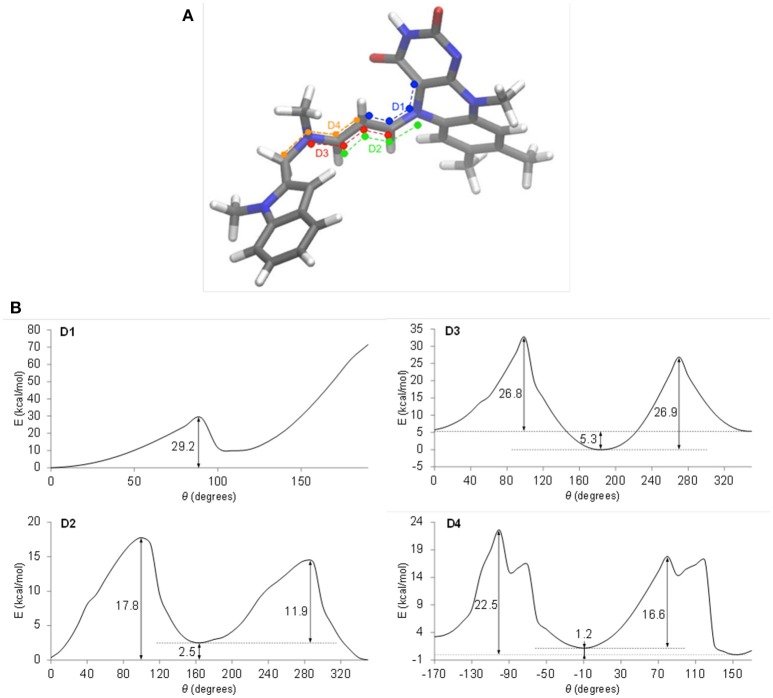
Energy profiles of internal rotations in the truncated model of the pentapeptide-FAD-ASS234 adduct. **(A)** The four dihedral angles are defined in blue (D1), green (D2), red (D3), and orange (D4). **(B)** Energy profiles of internal rotations around dihedral angles D1, D2, D3, and D4. Relaxed scans were performed on the M062x/6-311+G(d,p) level of theory.

To define which interconversions give the observed *cis-trans* isomerism, quantum chemical calculations of cyanine internal rotations were performed using a truncated model of the pentapeptide-FAD-ASS234 covalent adduct (Figure 5A). The energy profiles for the four dihedral angles D1–D4 in the cyanine chain were calculated (Figure 5B) and compared to our chromatographic experimental data obtained at 10°C. According to the D1 internal rotation energy profile (Figure 5B, D1) it is very unlikely that this rotation is responsible for the doubled peaks in the chromatogram. However, the system is at a low energy state at θ = 0° where the cyanine structural element is co-planar and in close proximity to the pyrimidinedione ring of isoalloxazine moiety. This indicates that the steric hindrance imparted by the carbonyl oxygen is compensated by the mutual electrostatic stabilization of the positive charge (cyanine chain) and negative charge (pyrimidinedione ring in FADH^−^); other flavoprotein oxidases, but not MAO, have a positively charged amino acid near N(1) to stabilize the anionic hydroquinone (Ghisla and Massey, 1986; Edmondson et al., 2004a). An energy increase at θ ≈ 90° is indicative of the disruption of conjugation, but as the dihedral angle approaches 180° and the positive charge moves further away from the pyrimidinedione ring, the electrostatic stabilization weakens, evidenced by an unfavorable and substantial increase in energy (>70 kcal/mol). Dihedral angles of >180° also resulted in steric repulsion, giving rise to very high energies and, thus, the geometry optimization procedure was automatically terminated at this point.

The energy profile for D2 (Figure 5B, D2) shows asymmetry of 2.5 kcal/mol, which is in a relatively good accordance with the experimental value of 1 kcal/mol, but energy barriers of 11.9 and 17.8 kcal/mol should produce a single peak in the chromatogram at 10°C as a result of rapid interconversion between isomers. Considering the D2 rotation further, the system is most stable at θ = 0°, which puts the ASS234 nitrogen in the cyanine chain in close proximity to the pyrimidinedione ring. This prevents the localization of the positive charge on the N(5) nitrogen and enables optimum electrostatic and resonance energy stabilization of the adduct. Nonetheless, the energy difference of the system at θ = 0° and θ ≈ 180° is negligible compared to the D1 rotation.

The activation energies (26.8–26.9 kcal/mol) for the internal rotation around D3 (Figure 5B, D3) are too high to explain the phenomena in the chromatograms, but the large asymmetry of 5.3 kcal/mol indicates that the system clearly favors the ASS234 tail turned away from the FAD at θ ≈ 180°.

Finally, the barrier of 22.5 kcal/mol shown in the profile of D4 (Figure 5B, D4) and the energy asymmetry of 1.2 kcal/mol are in excellent agreement with the experimentally determined values of 21.3 kcal/mol (DCXplorer) and 1 kcal/mol, respectively. Profile D4 is further discussed in Discussion S3. These results indicate that geometric isomerization about this C–N bond (D4 internal rotation) is most likely responsible for the two isomers observed in the chromatogram at 10°C, while the second pair of peaks appearing at −10°C could be attributable to isomerization about the C–C bond (D2). Relative ease of rotation around D2 and D4 in comparison to D1 and D3 also suggests that the positive charge is not as evenly delocalized in the cyanine chain as initially suspected, but is preferentially concentrated at N(5) due to the strong electrostatic stabilizing effect. What is more, these energy profiles indicate that the electrostatic stabilization of the adduct is far greater than stabilization through charge delocalization.

**Figure 6:**
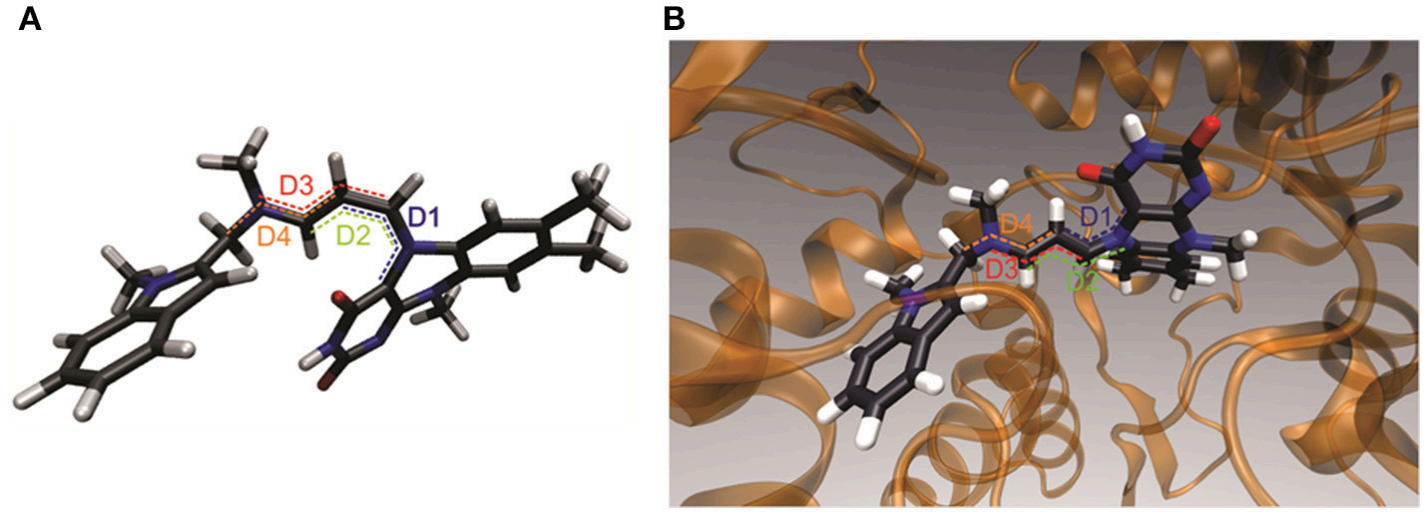
A 3D structural representation of energetically most stable geometric adduct structure which has the largest contribution to the overall compound configuration. **(A)** Free truncated adduct. **(B)** Adduct inside the active site of MAO A.

These quantum chemical calculations considered each internal rotation individually in order to obtain energy profiles. However, when a minimum potential energy of truncated adduct was calculated by full geometry optimization in four-dimensional space (starting from the above optimal values of dihedral angles, D1 = 0°, D2 = 350°, D3 = 180°, and D4 = 150°), slightly different cyanine chain dihedral angles were obtained (D1 = 312°, D2 = 353°, D3 = 183°, and D4 = 94°). The general molecule configuration stays relatively preserved and the cyanine chain is slightly coiled. This puts the cyanine CH_2_ group (next to the tail end tertiary amine) below the bent plane of the isoalloxazine ring, thus, avoiding any steric hindrance with the carbonyl group of the pyrimidinedione ring (Figure 6A). The O … C distance of 2.8 A implies a slight attraction which could be attributed to a CH … O quasi-hydrogen bond or electrostatic attraction of the positively charged cyanine chain and negatively charged pyrimidinedione ring. The four consecutive C–N and C–C bond lengths in the chain, starting from the N(5) atom of the flavin, are 1.32, 1.40, 1.38, and 1.33 Å. These support a partial double bond character and at the same time give an additional evidence of an unequal delocalization of the positive charge, which is slightly more concentrated at the N(5) end of the cyanine chain.

Based on the results from the experiments and the quantum chemical calculations, the most stable structure for the FAD-containing pentapeptide-ASS234 covalent adduct is that shown in Figure 6A. Configuration of the C–N(5) bond is rigid, extending outwards below the plane of the middle flavin ring with the cyanine chain bent toward the electron rich pyrimidinedione ring. Although the rest of the conjugated chain has a higher degree of flexibility, a quasi-planar cyanine chain configuration is favored because it enables resonance stabilization of the positive charge. The cyanine chain breaks from the all-*trans* configuration and reorients to maximize electrostatic stabilization with the pyrimidinedione ring. Although the complexity of the adduct can certainly not be captured with a single structural representation, we conclude that this geometric structure has the largest contribution to the overall compound configuration.

However, docking the truncated adduct into the MAO A active site revealed that the ideal adduct structure cannot be developed in the active site. Here, the environment of the enzyme does not support a coiled cyanine structure and instead, a more extended chain is demonstrated to be most energetically favorable (Figure 6B). However, the charged chain remains bent toward pyrimidinedione ring which again demonstrates the significance of stabilization by attraction of opposite charges. This configuration of a quasi-all-*trans* conjugated chain is in good accordance with the electron density in the crystal structure of the intact MAO B-ASS234 adduct reported by Esteban et al. (2014). Their X-ray data revealed the position of the inhibitor inside the active site but at 1.8 Å resolution did not necessarily provide enough information to deduce the detailed structure of the adduct. The structure and data in our study allow for quantum chemical calculations. The resulting structure is consistent with the abovementioned electron density map but provides more detail (such as position of hydrogen atoms and saturation degree of aliphatic linker) and is more relevant to the enzyme in the cell rather than in a fixed crystal. A neutral pyrimidinedione ring and the lack of a cyanine moiety in the adduct structure proposed by Esteban et al. does not allow for isomerism observed in our study because rotations about single bond or double bonds in the adduct linker are believed to be unhindered or energetically extremely unfavorable, respectively. Our study also confirms that the whole ASS234 is attached to FAD as was implied by the intact mass but not visible in the crystal (Esteban et al., 2014).

### Chemical mechanism of MAO-A irreversible inhibition by ASS234

**Figure 7:**
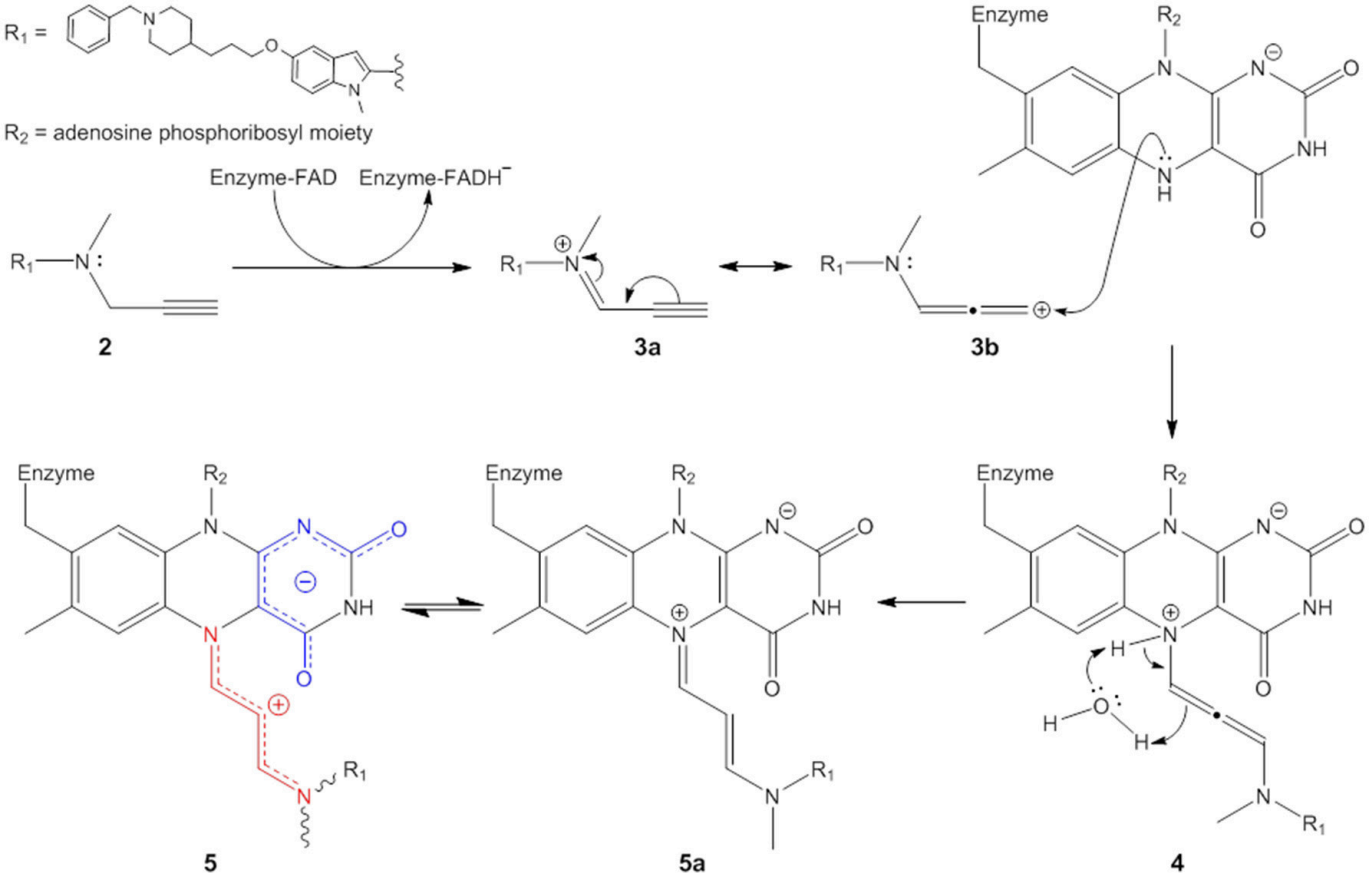
Proposed mechanism of irreversible MAO inhibition by propargylamines.

Based on the results of this study, we propose a new chemical mechanism for MAO-A inactivation by ASS234 that is consistent with all published experimental data (Figure 7). This pathway could be extended to both MAO enzymes and propargylamine inhibitors in general.

The propargylamine inhibitors (**2**) are substrate analogs that can be processed by the targeted enzyme to generate highly reactive species which in turn covalently modify the enzyme and suppress its catalytic activity (Silverman, 1995a; Szewczuk et al., 2007). The first step is ASS234 oxidation by MAO giving the iminium cation (represented here by two resonance forms **3a** and **3b**) and FADH^−^ (see further reasoning in Discussion S4). The mechanistic pathway of this reaction has been extensively reviewed (Chajkowski-Scarry and Rimoldi, 2014). Although the imine is highly reactive, hydrolysis measured for a MAO–B imine product is slower than catalytic turnover indicating that hydrolysis to aldehyde happens in the solvent after dissociation (Edmondson et al., 1993; Woo and Silverman, 1995).

One important question arises. Why do oxidized substrate amines leave the enzyme as oxidation products but these inactivators do not? It has been shown that positively charged molecules can be trapped by favorable cation-π interactions in the active site “aromatic cage” formed by aromatic amino acid residues Tyr^406^ and Tyr^444^, and the isoalloxazine ring (Borštnar et al., 2012). Our results show that the attraction between a positively charged species and negatively charged pyrimidinedione ring also presents a considerable electrostatic force. Therefore, prior to dissociation the charged oxidized substrates are deprotonated in a concerted reaction involving nearby water molecules (Vianello et al., 2012). On the other hand, many well-known tertiary amino substrates such as MPTP and hordenine (*N,N*-dimethyltyramine) are poor MAO substrates. MAO oxidation products of these species are also represented by charged imines, but these bear no hydrogens on the quaternary nitrogen atom. The deprotonation and therefore neutralization cannot occur and the disengagement of these molecules from MAO active site is hindered due to the strong electrostatic forces. ASS234 and the other propargylamines also belong to the group of tertiary amines. Oxidation of C–N bond in the beta position to the alkynyl functional group in ASS234, gives a charged species (**3a**,**3b**) which is unable to dissociate from MAO-A active site. Our reasoning is supported by a previous study which showed that methylation of the amino group significantly increased the activity of a propargylamine inhibitor whereas species containing -NH- were less efficacious (Kalir et al., 1981). The subsequent nucleophilic attack of FADH^−^ is thermodynamically driven as the adduct structure clearly demonstrates the allenyl resonance form (**3b**) as being involved in the reaction (see Discussion S5 for further explanation). The kinetic product proceeding via nucleophilic attack of FADH^−^ on the ASS234 carbon in the alpha position to the alkynyl functional group (**3a**) would be represented by a highly branched compound suffering from severe steric hindrance and would lack the observed UV-VIS distinctive chromophore at λ_max_ = 410 nm. The outcome of the nucleophilic addition is a substituted 1,3-diaminoallene (**4**). This allene undergoes a rapid 1,3-prototropic rearrangement, as was shown for similar systems (Maas and Mayer, 1991; Espenlaub et al., 2007). The reaction is assumed to be mediated by a nearby water molecule which indirectly transfers the proton from N(5) nitrogen to the sp hybridized allenyl carbon atom, which eventually yields (**5**). Consequently, the positive charge can be delocalized along the newly formed cyanine chain that preferentially adopts a coiled conformation to allow maximum electrostatic stabilization.

## Conclusions

MAO-A unambiguously forms a covalent adduct with the propargylamine inhibitor ASS234. The bond between the two species was shown here to comprise of a cyanine linker. This electron deficient moiety has a double bond character which gives rise to an intricate structural equilibrium of the adduct, resulting in geometric isomerism of the inactivated flavopentapeptide. The most stable coiled isomer of the adduct revealed that the intramolecular electrostatic interactions are of paramount importance in terms of compound stability. Additionally, the flexibility of the inhibitor to adopt different configurations is important as it prevents steric hindrances between the positively charged cyanine linker and the electron rich pyrimidinedione ring of FAD and finally, between ASS234 and the immediate surroundings in the MAO-A active site when the whole system is considered. The mutual electrostatic attraction of two opposing charges seen here has important implications for the catalytic mechanism of MAO in that oxidized charged substrates are not likely to dissociate from the enzyme active site until they are deprotonated or the pyrimidinedione ring is neutralized. In addition, the anionic hydroquinone, detected here in the final adduct, might have a bigger role in inactivation and catalysis than previously recognized. Delocalized electronic density which spreads over the entire pyrimidinedione ring in reduced MAO could facilitate (re)orientation of charged and polarizable products. Further experiments, which are outside of the scope of this study, are needed to confirm these hypotheses.

Based on the results presented here, and in correlation with published experimental data, the full inhibition mechanism of MAO-A by propargylamines is proposed. The mechanism described here is important both from theoretical and practical standpoints. As well as a relevant foundation for mechanistic studies of other flavoenzymes, our findings present information for the rational design of efficient new generation drugs for the treatment of neurodegenerative and neuropsychiatric disorders.

## Author contributions

AA, JM, and RRR conceived the study and wrote the draft manuscript. JMC designed and synthesised the ASS234. AA and RRR performed the biological studies. AA and IV performed and interpreted the chemical analyses. JM performed quantum chemical calculations and analyzed the results. All authors revised and approved the final manuscript.

### Conflict of interest statement

The authors declare that the research was conducted in the absence of any commercial or financial relationships that could be construed as a potential conflict of interest.

## Supplementary material

Full experimental details, supporting figures, and supporting discussions (PDF). This material is available free of charge online.
