# Supplementary material for: Evidence for a Cyanine Link Between Propargylamine Drugs and Monoamine Oxidase Clarifies the Inactivation Mechanism
Source: Front Chem. 2018 May 28;6:169. doi: 10.3389/fchem.2018.00169 (PMC5985292; doi:10.3389/fchem.2018.00169)
Supplement: Supplementary file 1 [file Data_Sheet_1.docx]

**Evidence for a Cyanine Link between Propargylamine Drugs and Monoamine Oxidase Clarifies the Inactivation Mechanism**

**Alen Albreht,^1,*^ Irena Vovk,^1^ Janez Mavri,^2^ Jose Marco-Contelles,^3^ Rona R. Ramsay^4,*^**

^1^ National Institute of Chemistry, Department of Food Chemistry, Ljubljana, Slovenia

^2^ Laboratory of Computational Biochemistry and Drug Design, National Institute of Chemistry, Theory Department, Ljubljana, Slovenia

^3^ Laboratorio de Química Médica, Instituto de Química Orgánica General, CSIC and Centro de Química Orgánica “Lora-Tamayo”, CSIC, Madrid, Spain

^4^ Biomedical Sciences Research Complex, University of St Andrews, St Andrews, UK

**^*^ Correspondence:**

Dr. Alen Albreht Prof. Rona R. Ramsay

alen.albreht@ki.si rrr@st-andrews.ac.uk

**TABLE OF CONTENTS**

[1. MATERIALS AND METHODS 3](#_Toc506640929)

[1.2. Stopped-flow spectroscopy 3](#_Toc506640930)

[1.3. UV-VIS spectroscopy 3](#_Toc506640931)

[1.4. UHPLC-PDA-MS^n^ analyses 3](#_Toc506640932)

[1.4.1. Analysis of untreated and ASS234-inhibited MAO-A digests 4](#_Toc506640933)

[1.4.2. Detection of geometric isomers 4](#_Toc506640934)

[1.5. Computational methods 4](#_Toc506640935)

[1.6. Quantum chemical calculations of internal rotations of cyanine chain 5](#_Toc506640936)

[2. SUPPLEMENTARY FIGURES 6](#_Toc506640937)

[Figure S1. 6](#_Toc506640938)

[Figure S2. 7](#_Toc506640939)

[Figure S3. 8](#_Toc506640940)

[Figure S4. 8](#_Toc506640941)

[Figure S5. 9](#_Toc506640942)

[Figure S6. 10](#_Toc506640943)

[Figure S7. 10](#_Toc506640944)

[3. SUPPLEMENTARY DISCUSSION 11](#_Toc506640945)

[Discussion S1. 11](#_Toc506640946)

[Discussion S2. 11](#_Toc506640947)

[Discussion S3. 12](#_Toc506640948)

[Discussion S4. 12](#_Toc506640949)

[Discussion S5. 13](#_Toc506640950)

[4. REFERENCES 13](#_Toc506640951)

1. MATERIALS AND METHODS
   1. **Chemicals and materials**

MilliQ water (18.2 MΩcm^-1^) was acquired from a Milli-Q Gradient water purification system (Merck Millipore, Billerica, MA, USA), formic acid (HCOOH), LC-MS grade acetonitrile (MeCN), potassium hydroxide, potassium dihydrogen and hydrogen phosphate, ortho-phosphoric acid (85 %), hydrochloric acid (37 %), ammonia (25%), dimethyl sulfoxide, sodium chloride, calcium chloride and D-Tube Dialyzers were purchased from Merck Millipore and hydrophilic poly(vinyldiene difluoride) (PVDF) membrane filters (0.45 μm) from Millipore Millex-HV (Middlesex, USA). Trypsin and chymotrypsin were acquired from Promega (Madison, WI, USA) and phospholipase A and C, clorgyline hydrochloride, HEPES, Tris base, ethylenediaminetetraacetic acid (EDTA), dithiothreitol (DTT), phenylmethanesulfonyl fluoride (PMSF), d-amphetamine, triethylamine (TEA), Triton X-100, glycerol and β-octylglucoside were purchased from Sigma-Aldrich (UK). Human MAO-A expressed in yeast cells (*Saccharomyces cerevisiae*) was purified as before^1,2^ and stored at around 50 µM in 50% (v/v) glycerol/50 mM potassium phosphate buffer pH 7.0 at -20 °C. The propargylamine inhibitor ASS234 was synthesised as described previously^3^. ASS234 (10 mM) and clorgyline (5 mM) stock solutions were prepared in DMSO and kept at 4 °C.

- 1. Stopped-flow spectroscopy

Purified human MAO-A was dialyzed against 50 mM HEPES pH 7.5 for 3 hours to remove the D-amphetamine and glycerol used in the storage buffer and concentrated to at least 40 μM. The concentration of the purified MAO-A was determined by the substrate-induced absorbance change at 456 nm using the extinction coefficient^2^ of 10,800 M^-1^cm^-1^. MAO-A (40 μM) was mixed with an equal volume of 50 mM HEPES pH 7.5 containing ASS234 (120 μM) in the stopped-flow spectrophotometer (Applied Photophysics SX20) at 30°C. Absorbance changes were measured in triplicate runs at three wavelengths (410, 456, and 495 nm). The absorbance acquisition rate was set at 1000 Hz for the interval 0 – 0.5 s and 4 Hz for the interval 0.5 – 1000 s. The absorbance changes were analysed using single or double exponential fits in the built-in software as appropriate.

- 1. UV-VIS spectroscopy

Spectra in the range 300 – 600 nm were collected in Shimadzu 2101PC (Kyoto, Japan) and PerkinElmer Lambda 45 (Waltham, MA, USA) UV-VIS spectrophotometers in 50 mM HEPES pH 7.5 in quartz cuvettes with a 1 cm optical path length. To compare the absorption spectra of the inactivated FAD moiety at pH 1 and physiological conditions, the inhibited MAO-A digest was diluted 20-fold with 0.1 M hydrochloric acid or water, respectively, before acquisition of spectra.

- 1. UHPLC-PDA-MS^n^ analyses

The Thermo Finnigan Accela 1250 UHPLC system (San Jose, CA, USA) consisted of a thermostated Accela autosampler with a 25 μL loop, a quaternary high-pressure Accela pump, and a diode array detector Accela PDA detector. The UHPLC system was coupled to the LTQ Velos MS system (Thermo Finnigan) equipped with a heated electrospray ionization source (H-ESI). Xcalibur (2.1) software was used for evaluation of the collected data. All digest samples were diluted 10-fold with the mobile phase and filtered through a 0.45 μm PVDF filter prior to chromatographic analysis.

- - 1. Analysis of untreated and ASS234-inhibited MAO-A digests

The separations were carried out on an ACE Excel 2 μm C18 column (100 mm x 2.1 mm i.d.) from ACT (Aberdeen, UK). Flow rate was set to 300 μL/min, temperature was set to 30 °C (unless stated otherwise), absorption spectra were collected in the range 250 – 600 nm and two additional discrete channels were set at 410 nm and 450 nm. The injection volume was set to 5 μL (unless stated otherwise). The mobile phase consisted of solvent A (1 % HCOOH) and solvent B (MeCN + 1 % HCOOH). For the MAO-A digest the following gradient was used: 5 – 25 % B (0 – 14 min), 25 – 80 % B (14 – 14.5 min), 80 % B (14.5 – 16.5 min), 80 – 5 % B (16.5 – 17 min), 5 % B (17 – 24 min). For the MAO-A-ASS234 digest the gradient was adjusted to: 20 – 30 % B (0 – 14 min), 30 – 80 % B (14 – 14.5 min), 80 % B (14.5 – 16.5 min), 80 – 20 % B (16.5 – 17 min), 20 % B (17 – 24 min).

Mass spectra were acquired using ESI in the negative polarity mode. The acquisition of MS data was delayed for 2.6 min in each run to avoid excessive contamination of the MS source. The parameters of the MS source were set as follows: probe heater temperature = 200 °C, transfer capillary temperature = 275 °C, sheath gas = 25 arbitrary units (a.u.), auxiliary gas = 5 a.u., sweep gas = 0 a.u., spray voltage = 1.9 kV, and S-lens RF level = 66 %. For MAO-A digest analyses the MS scan range was set to *m/z* 400 – 1400 and MS^2^ was performed by fragmenting the precursor ion at *m/z* 1267 and then scanning the product ions in the range *m/z* 250 – 1400. The isolation width was set to 2 Da and normalized collision energy of 35 % was used. For MAO-A-ASS234 digest analyses the MS scan range was set to *m/z* 400 – 2000 and MS^2^ was performed by fragmenting the precursor ion at *m/z* 1710 and then scanning the product ions in the range *m/z* 470 – 2000. The isolation width was set to 3 Da and normalized collision energy of 35 % was used.

- - 1. Detection of geometric isomers

Chromatographic and MS parameters were the same as for the MAO-A-ASS234 digest analyses, only the temperature varied between 10, 30, 45, 60 and 75 °C. For the analysis at -10 °C, the column was removed from the Accela oven compartment and was submerged in a cooling bath consisting of ice, water, and sodium chloride. The flow rate was set at 200 μL/min and the following gradient was used: 25 – 35 % B (0 – 19 min), 35 – 80 % B (19 – 19.5 min), 80 % B (19.5 – 22.5 min), 80 – 25 % B (22.5 – 23 min), 25 % B (23 – 30 min).

The fractionation of 2 isomers was achieved at 10 °C by injecting 25 μL of MAO-A-ASS234 digest solution onto the column and collecting each individual peak separately at the PDA exit. The process was repeated 5 times. The total volume of both fractions was brought down to 50 μL and 25 μL of each fraction was analysed separately by UHPLC at 30 °C.

- 1. Computational methods

The Gibbs activation energy ($\Delta G^{\ddagger}$) for the *cis-trans* isomerization of the cyanine chain in the adduct between ASS234 and FAD moiety of MAO-A was determined from experimental chromatographic data by employing the transition state theory. The interconversion energy barrier was calculated by using the Eyring-Polanyi equation, which describes the relation between the reaction rate and the activation free energy:

$$k=\frac{k_{B}T}{h}e^{-\frac{\Delta G^{\ddagger}}{RT}}$$

where *k* = reaction rate constant, *k_B_* = Boltzmann constant, *h* = Planck’s constant, *T* = temperature, and *R* = gas constant. By determination of the reaction rate constant at a given temperature the Gibbs activation energy for the reaction was calculated. Rate constants for the isomerization observed in chromatograms at a particular temperature were determined by using DCXplorer software developed by Trapp^4^, which uses the unified equation to evaluate elution profiles of reversible first-order reactions in dynamic chromatography and on-column reaction chromatography. Rate constants and, consequently, Gibbs activation energies were determined by processing the experimental chromatographic data at 10 °C and 30 °C.

- 1. Quantum chemical calculations of internal rotations of cyanine chain

Initial structure of the studied pentapeptide-FAD-ASS234 adduct was obtained by model building using the MOLDEN program^5^. To simplify the simulations a truncated model of the adduct was used since adenosinephosphoribityl and pentapeptide moieties as well as the cholinesterase inhibiting part of ASS234 were assumed to have no significant contribution on the configuration of the cyanine chain. Four internal rotations were considered and energy profiles were calculated by using a relaxed scan. The rationale behind the relaxed scan is that for each value of dihedral angle all other degrees of freedom were optimized. The starting geometry for each dihedral angle was the optimized geometry of preceding dihedral value. Calculations were performed on the M062x/6-311+G (d,p) level. The applied level of theory represents a compromise between the available CPU power and size of the systems. The M062X functional was designed by Truhlar's group to reproduce thermodynamic and kinetic parameters, being particularly successful in treating nonbonding interactions, and not underestimating barrier like many other density functional theory approaches. Calculations of some selected stationary points with included solvent reaction field have shown that the effect of solvent on the free energy profiles was minor. Therefore all the reported results are for the gas phase and they correspond also to the complex in solution. All calculations were performed using the Gaussian 09 suite of programs^6^.

1. SUPPLEMENTARY FIGURES

Figure S1.

UV-VIS spectrum of MAO-A-ASS234 adduct at pH 7.5 (dotted line) and at pH 1 (solid line). The absorption maximum is shifted to 390 nm in acidic medium which is in line with the covalent attachment of ASS234 at N(5) position of FAD.

Figure S2.

UHPLC chromatogram of tryptic/chymotryptic digest of MAO-A (acquired at 450 nm). A single peak in the chromatogram corresponds to the pentapeptide-FAD.

Figure S3.

MS spectrum of pentapeptide-FAD (chromatographic peak at 6.9 min). Signals for the singly deprotonated (*m/z* 1267) and doubly deprotonated (*m/z* 633) molecules are observed.

Figure S4.

A single fragment ion in MS^2^ spectrum of pentapeptide-FAD (chromatographic peak at 6.9 min, precursor ion at *m/z* 1267). The fragment ion at m/z 920 is the result of a neutral loss of 347 – adenosine monophosphate (AMP).

Figure S5.

A mass spectrum of the pentapeptide-FAD-ASS234 covalent adduct (chromatographic peak at 7.4 and 8.8 min).

Figure S6.

MS^2^ spectrum of the pentapeptide-FAD-ASS234 covalent adduct (chromatographic peak at 7.4 and 8.8 min, precursor ion at *m/z* 1710). The fragment ion at m/z 1363 is the result of a neutral loss of 347 – adenosine monophosphate (AMP).

Figure S7.

A chromatogram of tryptic/chymotryptic digest of clorgyline inhibited MAO-A acquired at 410 nm (T = 45 °C). Two isomers at 5.9 and 9.1 min are well resolved even at an elevated temperature due to a higher interconversion energy barrier.

1. SUPPLEMENTARY DISCUSSION

Discussion S1.

**Tailing/fronting of chromatographic peaks in the chromatogram of ASS234 inactivated MAO-A digest (elevated baseline between peaks)**

Tailing and fronting of chromatographic peaks is quite a common phenomenon in chromatography when the analyte exhibits some secondary interactions with the stationary phase or material surfaces, or when the LC column is overloaded with the sample. Phosphate functional groups of FAD represent good chelating agents and they can interact with trace amounts of metal ions, which can be found in stainless steel parts of a non-biocompatible LC system or in low purity type silica stationary phases. In our case, the addition of EDTA to the injection solvent, which usually masks these metal ions, did not improve the peak shape of the two peaks in question. Moreover, should phosphate groups of FAD be the cause of these peak asymmetries, this would have also been observed in the untreated MAO-A digest. The acidic pH of the mobile phase (pH ~ 2) ensured that the silanol interactions did not contribute to this anomaly either. Decreasing the injection volume also had no effect on the shape of these chromatographic peaks, ruling out column overload. Therefore, all typical sources of artefacts were systematically ruled out so the bridging between the two peaks in the chromatogram of ASS234 inactivated MAO-A digest was shown to be a consequence of an intrinsic structural feature of the adduct after inactivation of MAO-A by ASS234.

Discussion S2.

**Covalent bond formation between MAO-A and ASS234 is supported by MS data**

The mass spectra obtained in the LC-MS analyses have a high background primarily due to a high formic acid concentration in the mobile phase which was crucial to achieve a good chromatographic separation. An additional contribution to the busy MS background was made by the heavy sample matrix. This included surfactants, enzyme stabilizers, non-FAD containing peptides, etc. which co-eluted within the retention time frame of pentapeptide-FAD-ASS234. However, the observed mass of ASS234 modified pentapeptide-FAD unambiguously confirms that a covalent bond is formed between the inhibitor and MAO-A. The obtained experimental isotopic distribution of adduct signal at *m/z* 1710 is in good accordance with the theoretical distribution (Table 1).

Table 1: Isotope distribution for C_75_H_95_O_24_N_17_S_1_P_2_. The experimental values are given as the average relative abundances across the chromatographic peak of the adduct.

| ***m/z*** | **Relative abundance** | |
| --- | --- | --- |
|  | **Theoretical** | **Experimental** |
| 1710.5859 | 100 | 100 |
| 1711.5883 | 91.50 | 92.55 |
| 1712.5906 | 50.75 | 50.03 |
| 1713.5930 | 20.87 | 20.77 |
| 1714.5953 | 6.92 | 5.54 |

The formation of a covalent bond is not always clear-cut unless the protein is unfolded and digested, as done here. Bulkier propargylamine inhibitors can sometimes act as a plug and inactivate MAO by preventing access to the active site^7^. A direct MS analysis of the intact inhibited enzyme (non-digested) or prolonged dialysis may not be sufficient to indicate the type of interaction between the enzyme and the inhibitor in these cases.

Discussion S3.

**Local minima observed in the energy profile of D4 internal rotation**

An interesting feature is seen in the energy profile of D4 at roughly *θ* = 90° and *θ* = -90°, which is not observed in other profiles. At these dihedral angles p-orbital on the carbon and the lone pair orbital on the nitrogen are perpendicular to each other. Consequently, the nitrogen cannot donate its lone electrons to the system, which reduces the cyanine chain resonance. This should be evidenced as an increase in the energy profile, but on the contrary, there are two local minima observed which indicates a small relaxation of the system. This could be explained by a reduced steric hindrance imparted by the methyl group and ASS234 tail, but a more probable reason is that in this instance the electron deficiency (positive charge) is confined to a smaller segment within the cyanine chain and is therefore greater in magnitude which results in a small stabilizing electrostatic interaction with the pyrimidinedione moiety.

Discussion S4.

**Elucidation of the first step in the inactivation process of MAO by propargylamines**

It has previously been suggested that tertiary amine substrates, which includes propargylamines, inhibit MAO by forming a covalent intermediate during the course of a normal catalytic cycle. This intermediate is believed to be stable and does not proceed to form the final products (oxidized amine and reduced FAD), which in turn inhibits the enzyme’s activity^8,9^. However, our experimental data clearly show the existence of the cyanine link between MAO-A and ASS234, which could not have been formed if the inhibitor was not successfully oxidised by MAO-A in the first place (detailed explanation below). Additionally, the reaction leading to such inhibitory intermediates should be reversible and initial species of lower energy state (FAD and tertiary amine) should be observed after disruption of the catalytic site of inactivated MAO by proteolysis, but this was not observed in our study. It is also possible that different tertiary amines are not treated by MAO in the same mechanistic way, but this is very unlikely.

The ASS234 inhibitor is inactive until MAO acts upon it. Therefore, the oxidation of ASS234 by MAO must be the first step of the inactivation process in which the iminium cation and FADH^–^ are formed. This reaction is substantiated by many facts and observations: a) propargylamines possess the required structural features of MAO substrates, which are two hydrogen atoms on the α-carbon with respect to the amino functional group. Only one or no hydrogen atoms at this position results in competitive inhibition^10–12^; b) at first, propargylamine analogues act as concentration dependent inhibitors. The simultaneous presence of a substrate molecule prevents enzyme inactivation due to competition for the active site inside the enzyme^13–15^; c) propargylamines fail to inhibit reduced MAO in the absence of oxygen, which confirms that oxidized FAD is initially reduced by the inactivator^16^; d) free oxidized FAD in aqueous solution does not react with ASS234 (data not shown) but needs to be within the native MAO-A interior in order for the reaction to take place; e) the stopped-flow experiments show that the reduction of MAO-A precedes MAO-A-ASS234 adduct formation.

The results of quantum mechanics calculations reported by Borštnar *et al.* are in contradiction with the above findings; it was proposed that the first step in the inhibition pathway of MAO-A by propargylamines proceeds via proton abstraction from the alkynyl functional group of the inhibitor^17^. Their mechanism is also called into question by the lack of a base in the vicinity of the active site which could be strong enough to promote the removal of a proton with a pKa of ca. 25^12,18,19^.

Discussion S5.

**Reacting form of the oxidized ASS234 species**

The stabilization of the positive charge in oxidized ASS234 is achieved through resonance.

It is crucial for the alkynyl functional group to be in β-position to the nitrogen, otherwise the molecule does not act as an irreversible MAO-A inactivator^20^. The activated ASS234 could also undergo intramolecular transformation into the smallest aromatic system – the cyclopropenyl cation.

The aromatic three-membered ring of carbon atoms is 25-35 kcal/mol lower in energy than its aliphatic analogue^21–23^. The amino group in ASS234 even further lowers the overall energy of the cyclic system by lone electron pair donation. Hence, aromatization would stabilize the unstable inhibitor, but the transformation from an open-chain to a cyclic cation is impeded by a substantial activation barrier of ca. 100 kcal/mol^22,24^. Moreover, the activation energy for the subsequent reaction between cyclic ASS234 cation and N(5)-flavin would probably also increase. For instance, the reactivity of some inert cyclopropenyl salts is very poor since they were shown to be stable at room temperature and could readily be isolated in crystal form^24,25^. Therefore, cyclopropenyl isomer could react with MAO-A, but reaction of activated ASS234 in its linear configuration is more probable.

The contribution of individual linear mesomeric forms to the overall molecular structure can be strongly substituent dependent^26–28^. In our case, the propargylium resonance form of ASS234 has a larger contribution than the allenylium form; in addition to the destabilizing effect, which stems from the increased s-character of the sp^2^ hybridized carbon in the allenyl cation (C_3_), the low electron density at the iminium carbon (C_1_) is compensated by lone electron pair donation from the neighbouring amino group^29^. However, although the propargylium resonance form is theoretically prevalent, the structure of the adduct clearly proves that FADH^–^ attacks the terminal carbon atom of activated ASS234.

1. REFERENCES

(1) Tan, A. K., Weyler, W., Salach, J. I., and Singer, T. P. (1991). Differences in substrate specificities of monoamine oxidase A from human liver and placenta. *Biochem. Biophys. Res. Commun.* 181, 1084–1088. doi: 10.1016/0006-291X(91)92048-O

(2) Weyler, W., and Salach, J. I. (1985). Purification and properties of mitochondrial monoamine oxidase type-A from human-placenta. *J. Biol. Chem.* 260, 13199–13207.

(3) Samadi, A., de los Rios, C., Bolea, I., Chioua, M., Iriepa, I., Moraleda, I., et al. (2012). Multipotent MAO and cholinesterase inhibitors for the treatment of Alzheimer's disease: synthesis, pharmacological analysis and molecular modeling of heterocyclic substituted alkyl and cycloalkyl propargyl amine. *Eur. J. Med. Chem.* 52, 251–262. doi: 10.1016/j.ejmech.2012.03.022

(4) Trapp, O. (2006). Unified equation for access to rate constants of first-order reactions in dynamic and on-column reaction chromatography. *Anal. Chem.* 78, 189–198. doi: 10.1021/ac051655r

(5) Schaftenaar, G., and Noordik, J. H. (2000). Molden: a pre- and post-processing program for molecular and electronic structures. *J. Comput.-Aided Mol. Design* 14, 123–134. doi: 10.1023/A:1008193805436

(6) Frisch, M. J., Trucks, G. W., Schlegel, H. B., Scuseria, G. E., Robb, M. A., Cheeseman, et al. (2009). J. R., Gaussian 09, revision D. 01; Gaussian Inc.:Wallingford, Ct, 2009.

(7) Zindo, F. T., Barber, Q. R., Joubert, J., Bergh, J. J., Petzer, J. P., and Malan, S. F. (2014). Polycyclic propargylamine and acetylene derivatives as multifunctional neuroprotective agents. *Eur. J. Med. Chem.* *80*, 122–134. doi: 10.1016/j.ejmech.2014.04.039

(8) Vianello, R., Repič, M., and Mavri, J. (2012). How are biogenic amines metabolized by monoamine oxidases? *Eur. J. Org. Chem.* 7057–7065. doi: 10.1002/ejoc.201201122

(9) Ding, C. Z., and Silverman, R. B. (1993). Transformation of heterocyclic reversible monoamine oxidase-B inactivators into irreversible inactivators by N-methylation. *J. Med. Chem.* 36, 3606–3610. doi: 10.1021/jm00075a015

(10) Kalgutkar, A. S., Dalvie, D. K., Castagnoli, N., Jr., and Taylor, T. J. (2001). Interactions of nitrogen-containing xenobiotics with monoamine oxidase (mao) isozymes A and B: SAR studies on MAO substrates and inhibitors. *Chem. Res. Toxicol.* 14, 1139–1162. doi: 10.1021/tx010073b

(11) Nandigama, R. K., Newton-Vinson, P., and Edmondson, D. E. (2002). Phentermine inhibition of recombinant human liver monoamine oxidases A and B. *Biochem. Pharmacol.* 63, 865–869. doi: 10.1016/S0006-2952(02)00840-7

(12) Dostert, P., O’Brien, E. M., Tipton, K. F., Meroni, M., Melloni, P., and Strolin Benedetti, M. (1992). Inhibition of monoamine oxidase by the R and S enantiomers of N[3-(2,4-dichlorophenoxy)propyl]-N-methyl-3-butyn-2-amine. *Eur. J. Med. Chem.* 27, 45–52. doi: 10.1016/0223-5234(92)90059-A

(13) Silverman, R. B. (1995). [10] Mechanism-Based Enzyme Inactivators. *Methods Enzymol.* 249, 240–283. doi: 10.1016/0076-6879(95)49038-8

(14) Maycock, A. L., Abeles, R. H., Salach, J. I., and Singer, T. P. (1976). The action of acetylenic inhibitors on mitochondrial monoamine oxidase: structure of the flavin site in the inhibited enzyme. *CIBA Found. Symp.* 39, 33–47. doi: 10.1002/9780470720219.ch3

(15) Williams, C. H., and Lawson, J. (1974). Monoamine oxidase–II Time-dependent inhibition by propargylamines. *Biochem. Pharmacol.* 23, 629–636. doi: 10.1016/0006-2952(74)90627-3

(16) Hellerman, L., and Erwin, V. G. (1968). Mitochondrial monoamine oxidase. II. Action of various inhibitors for the bovine kidney enzyme. Catalytic mechanism. *J. Biol. Chem.* 243, 5234–5243.

(17) Borštnar, R., Repič, M., Kržan, M., Mavri, J., and Vianello, R. (2011). Irreversible inhibition of monoamine oxidase B by the antiparkinsonian medicines rasagiline and selegiline: a computational study. *Eur. J. Org. Chem.* 6419–6433. doi: 10.1002/ejoc.201100873

(18) Binda, C., Newton-Vinson, P., Hubálek, F., Edmondson, D. E., and Mattevi, A. (2002). Structure of human monoamine oxidase B, a drug target for the treatment of neurological disorders. *Nat. Struct. Biol.* 9, 22–26. doi: 10.1038/nsb732

(19) Binda, C., Mattevi, A., and Edmondson, D. E. (2002). Structure-function relationships in flavoenzyme-dependent amine oxidations: A comparison of polyamine oxidase and monoamine oxidase. *J. Biol. Chem.* 277, 23973–23976. doi: 10.1074/jbc.R200005200

(20) Weinreb, O., Amit, T., Bar-Am, O., and Youdim, M. B. H. (2010). Rasagiline: A novel anti-Parkinsonian monoamine oxidase-B inhibitor with neuroprotective activity. *Prog. Neurobiol.* 92, 330–344. doi: 10.1016/j.pneurobio.2010.06.008

(21) Pachuau, Z., Kharnaior, K. S., and Duncan Lyngdoh, R. H. (2013). Isomerization of propargyl cation to cyclopropenyl cation: Mechanistic elucidations and effects of lone pair donors. *J. Chem. Sci.* 125, 365–378. doi: 10.1007/s12039-013-0391-0

(22) Hopkinson, A. C., and Lien, M. H. (1986). Theoretical study of monosubstituted propargyl and cyclopropenyl cations, C_3_H_2_X^+^, and the reaction of C_3_H^+^ with HX. *J. Am. Chem. Soc.* 108, 2843–2849. doi: 10.1021/ja00271a010

(23) Radom, L., Hariharan, P. C., Pople, J. A., and Schleyer, P. V. R. (1976). Molecular orbital theory of the electronic structure of organic compounds. XXII. Structures and stabilities of C_3_H_3_^+^ and C_3_H^+^ cations. *J. Am. Chem. Soc.* 98, 10–14. doi: 10.1021/ja00417a003

(24) Wong, M. W., and Radom, L. (1989). Multiply charged isoelectronic analogs of cyclopropenyl/propargyl cation: cyclic or open chain? *J. Am. Chem. Soc.* 111, 6976–6983. doi: 10.1021/ja00200a012

(25) Breslov, R., and Groves, J. T. (1970). Cyclopropenyl cation. Synthesis and characterization. *J. Am. Chem. Soc.* 92, 984–987. doi: 10.1021/ja00707a040

(26) Surya Prakash, G. K., Krishnamurthy, V. V., Olah, G. A., and Farnum, D. G. (1985). Application of the Gassman-Fentiman tool of increasing electron demand to the carbon-13 NMR spectroscopic study of 1-aryl-3-methylbut-2-enyl (allylic) and 2-arylpent-3-yn-2-yl (propargylic) cations. *J. Am. Chem. Soc.* 107, 3928–3935. doi: 10.1021/ja00299a030

(27) Shchukin, A. O., Vasil’ev, A. V., and Grinenko, E. V. (2010). Reactions of arylacetylenic compounds with arenes in the presence of aluminum halides. *Russ. J. Org. Chem.* 46, 82–97. doi: 10.1134/S1070428010010082

(28) Lalli, P. M., Corilo, Y. E., Abdelnur, P. V., Eberlin, M. N., and Laali, K. K. (2010). Intrinsic acidity and electrophilicity of gaseous propargyl/allenyl carbocations. *Org. Biomol. Chem.* 8, 2580–2585. doi: 10.1039/c001985b

(29) Maas, G., Würthwein, E.-U., Singer, B., Mayer, T., and Krauss, D. (1989). Propiniminium-Salze: Ambifunktionelle Reaktivität gegenüber S- und N-Nucleophilen. *Chem. Ber.* 122, 2311–2317. doi: 10.1002/cber.19891221218
